# Potential influencing factor on health-related quality of life in Japanese with knee osteoarthritis: the Locomotive syndrome and Health outcome in Aizu cohort Study (LOHAS)

**DOI:** 10.1186/s40634-023-00649-1

**Published:** 2023-08-26

**Authors:** Kenichi Otoshi, Shinichi Kikuchi, Koji Otani, Tatsuru Sonobe, Miho Sekiguchi, Shinichi Konno

**Affiliations:** 1 Department of Sports Medicine, Fukushima Medical University, 1 Hikarigaoka, Fukushima, 960-1295 Japan; 2 Department of Orthopaedic Surgery, Fukushima Medical University School of Medicine, Fukushima, Japan

**Keywords:** Knee osteoarthritis, Health-related quality of life, Influencing factor, Self-efficacy, Epidemiologic study

## Abstract

**Purpose:**

Several studies have investigated the factors that influence health-related quality of life in patients with knee osteoarthritis (KOA). This study aimed to identify and investigate the degree of involvement of potential factors influencing health-related quality of life (HRQOL) in an aged population with or without KOA.

**Methods:**

This multi-centered study included 651 participants who underwent health checkups in rural areas of Japan in 2010. The association between three component summary score of short-form 12 (physical component summary; PCS, mental component summary; MCS, and role-social component summary; RCS) and covariates were investigated using multiple linear regression model and calculated the scaled estimated regression coefficient.

**Results:**

Decreasing mobility, severity of knee pain, high pain-related self-efficacy (PSE), older age, high functional self-efficacy (FSE), and female gender had significant effect on PCS (*p* < 0.05). However, radiographic KOA had no influence on PCS. Presence of depression and body mass index had a significant influence on the MCS (*p* < 0.05). Decreasing mobility, presence of depression, PSE and older age had significant influence on the RCS (*p* < 0.05).

**Conclusion:**

Our study results showed that physical, mental, and role/social QOL were affected by different influencing factors. Physical QOL was strongly influenced by subjective pain, physical performance, and self-efficacy, whereas radiographic KOA had no such effect. Depressive mood is associated with both mental and role/social QOL. The role/social QOL was predominantly affected by physical function and pain-related self-efficacy. Taking measure to improving functional ability and mental status might be the key factor to improve HRQOL in patient with KOA.

**Level of evidence:**

Level 3: Epidemiologic cross-sectional study (prognostic study)

## Introduction

Knee osteoarthritis (KOA) is a major public health concern that causes chronic pain and disability in the older population. Morphological changes in the subchondral bone, articular cartilage degeneration, and damage to the surrounding soft tissue can cause knee pain, stiffness, and limited movement [23]. About 25-million subjects aged over 40 years are estimated to have osteoarthritic changes in their knee joints [45].

The fundamental purpose of KOA treatment is to prioritize pain control and improve function. Recent studies have highlighted the importance of the overall well-being of patients with KOA. It is evident that the presence of KOA has a significantly negative impact on health-related quality of life (HRQOL) [5, 21, 31, 41, 42, 44]. Most studies have shown that HRQOL is worse in patients with KOA than those without KOA [1, 10, 12, 21, 28, 36, 43]. Pain and physical dysfunction induced by KOA might have a harmful influence not only on physical disability, but also on psychological conditions and social activities, such as social connectedness and relationships.

Several studies have investigated factors influencing HRQOL in patients with KOA. Increasing age [7, 28, 30], female gender [14, 19], reduced physical activity [43], reduced lower extremity muscle power [33], psychological distress and depression [12], lower educational level [1], and lack of familial relationships [22] are the major presumed factors affecting HRQOL in patients with KOA. However, few studies have investigated the extent of the influence of these influencing factors on HRQOL in the elderly population using large epidemiological data. Furthermore, the impact of self-efficacy on HRQOL has been not well investigated. Therefore, this study aimed to identify and investigate the degree of involvement of potential influencing factors on HRQOL in older population with or without KOA using cross-sectional data from the Locomotive Syndrome and Health Outcome in Aizu Cohort Study (LOHAS) [29]. Our study hypothesis was that functional and emotional factors, including self-efficacy, might have a strong influence on HRQOL as well as radiographic severity in patient with KOA characteristics.

## Materials and methods

### Study participants

The study protocol was approved by the Ethics Committee of Fukushima Medical University and all participants provided written informed consent. This cross-sectional study used data from the Locomotive Syndrome and Health Outcomes in the Aizu Cohort Study (LOHAS). The LOHAS is a cohort study that began in 2008, involving residents aged 40–80 years who participated in annual health check-ups in two communities (Tadami and Minamiaizu) in Fukushima Prefecture, Japan [29]. Our study participants included adults aged > 40 years who underwent a health check and completed a questionnaire in 2009. Knee X-ray examination was on participants who opted for it as an optional extra of annual health check-ups. An anteroposterior weight-bearing knee X-ray was taken in the examination car on the day of annual health check-ups.

### Clinical outcomes

Health-related quality of life (HRQOL) is a multidimensional measure of subjective feelings and experiences with health, consisting of physical, mental, and social functioning. General HRQOL was assessed using the Medical Outcome Study Short Form 12-Item Health Survey (SF-12) [13] and the three summary scores for physical component summary (PCS), mental component summary (MCS), and role/social component summary (RCS) [39]. The PCS, which represent physical QOL, includes six subscales (physical functioning, bodily pain, general health, physical role, social functioning, and vitality); the MCS, which represent mental QOL, includes six subscales (bodily pain, general health, social functioning, emotional role, vitality, and mental health); and the RCS, which represent role/social QOL, includes five subscales (bodily pain, general health, physical role, social functioning, and emotional role).

### Exposure factors

We collected sociodemographic data including sex, age, and body mass index (BMI). We defined as “overweight” when a BMI was ≥ 25 kg/m^2^ based on the criteria of the Japan Society for the Study of Obesity [11]. Previous episodes of knee pain during the past 1-month were assessed using a self-completed questionnaire and classified into three grades: none, mild-to-moderate, and severe. Depressive symptoms were also assessed using the 10-item version of the Center for Epidemiological Studies Depression Screening Index (CES-D-10), designed to quantify the number and frequency of depressive symptoms. The cutoff score for depressive symptoms was defined as ≥ 10 for the 10-item version [2].

Pain related self-efficacy (PSE) and functional self-efficacy (FSE) were assessed using questions from the arthritis self-efficacy scale (ASES) developed by the Stanford Patient Education Resource Center [24]. The ASES consists of three subscales: management of pain, function, and other symptoms. We selected one question from the pain management subscale of the ASES and modified it to evaluate knee arthritic pain. Our modified questions were as follows: “How certain are you that you can keep your knee arthritis pain from interfering with your daily activity?”. The response are 0 = “not at all”; 1 = ”not have much”; 2 = ”moderately”; 3 = ”quite a bit”; 4 = ”extremely,” which were then divided into two grades: low PSE group (#0,1) and high PSE group (#2–4). We also selected one question from the functional management subscale of the ASES as follows: “How certain are you that you can get out of an armless chair quickly, without using your hands for support?”. The response are 0 = “not at all”; 1 = ”not have much”; 2 = ”moderately”; 3 = ”quite a bit”; 4 = ”extremely,” which were then divided into two grades: low FSE group (#0,1) and high FSE group (#2–4).

To assess mobility and quantify locomotor performance, timed up and go test (TUG-test) was conducted [32]. This test includes basic mobility skills such as rising from a chair, walking 3 m, turning, and sitting down on the same chair. Since it has been reported that an individual who performs the test in < 10 s (s) is regarded as a very dependent person who cannot transfer out of a chair or walk without assistance [32], we defined TUG test < 10 s as normal mobility.

The severity of radiographic KOA was graded based on the Kellgren and Lawrence (KL) radiographic grading system. Two well-trained knee surgeons independently assessed the anteroposterior view of the knee radiographs. intra-observer reliability was 0.653, and inter-observer reliabilities were 0.653 and 0.652, respectively. Since the reported intra- and inter observer reliabilities of the KL-grade classification were 0.56 and 0.61, respectively, our grading accuracy was either equal to or surpassed that of a previous report [15]. We classified the severity of KOA into three grades: none (KL0), mild to moderate (KL1,2), and severe (KL3,4). If the participants had bilateral differences in the severity of radiographic KOA, the grade of the more severe side was selected.

### Statistical analysis

Participants with complete data were included in statistical analyses. Descriptive statistics were calculated for participants’ baseline characteristics. Continuous data were summarized as means and standard deviations, while dichotomous or categorical data were provided as proportions.

One-way ANOVA was used to investigate the association between each HRQOL score and confounding factors. Furthermore, a multiple linear regression model was used to calculate the scaled estimated regression coefficient (β).

Variance inflation factor (VIF) is a measure of multicollinearity in a set of multiple regression variables. A high VIF indicates that the associated independent variable is highly collinear with other variables in the model.

All statistical analyses were conducted using the JMP software, version 15.0.0 (SAS Institute, Cary, NC, USA). All tests were two-sided, and *P*-values of < 0.05 were considered statistically significant.

## Results

**Fig. 1:**
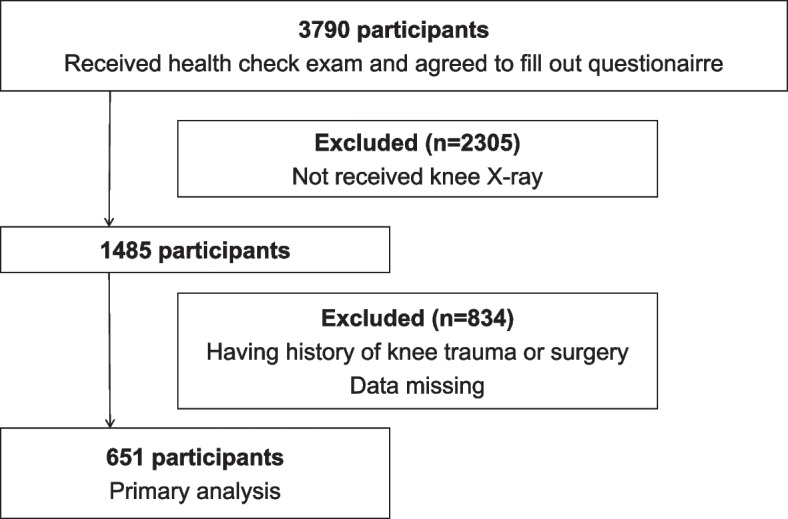
Study flowchart

The selection process for study participants is shown in Fig. 1. Among the 3,790 participants in LOHAS 2009, 1,485 underwent knee X-ray assessment. After excluding 186 participants with a history of knee trauma or surgery and 648 participants with missing data on confounding factors or outcome variables. Finally, 651 participants were included in the statistical analysis (Fig. 1).

The baseline characteristics of the 651 patients are presented in Table 1. The mean age of the participants was 69.5 (standard deviation; SD, 7.2) years, and 64.8% were female. The prevalence of radiographic KOA was 94.2% (614 subjects). In detail, the prevalence of mild to moderate KOA was 47.7% (311 participants) and that of severe KOA was 46.5% (303 participants). The 96.5% of the participants experienced knee pain during the past 1 month (mild to moderate pain, 82.7%; severe pain, 13.8%). The average time taken for the TUG test was 8.1 s (SD, 2.7). The prevalence of participants with decreasing mobility (TUG test≧10 s) was 15.2%.

**Table 1:** Table baseline characteristics of 651 participants

age(years)	mean ± SD	69.5 ± 7.2
	< 50	12(1.8%)
	50–59	40(61.4%)
	60–69	244(37.5%)
	70≦	355(54.5%)
gender	male	229(35.2%)
	female	422(64.8%)
BMI(kg/m2)	mean ± SD	24.4 ± 3.1
	BMI < 25	390(60.0%)
	BMI≧25	261(40.0%)
radiographic KOA	none	38(5.8%)
	mild-moderate	311(47.7%)
	severe	303(46.5%)
knee pain	none	23(3.5%)
	mild-moderate	539(82.7%)
	severe	90(13.8%)
TUG(sec)	mean ± SD	8.1 ± 2.7
	TUG < 10	552(84.8%)
	TUG≧10	99(15.2%)
CES-D-10(point)	mean ± SD	5.6 ± 4.4
	CES-D-10 < 9	545(83.7%)
	CES-D-10≧10	106(16.3%)
PSE(point)	mean ± SD	2.8 ± 0.8
	low PSE	266(41.0%)
	high PSE	385(59.0%)
FSE(point)	mean ± SD	3.4 ± 0.9
	low FSE	100(15.4%)
	high FSE	551(84.6%)
PCS (point)	mean ± SD	41.6 ± 14.0
MCS (point)	mean ± SD	50.6 ± 10.2
RCS (point	mean ± SD	48.0 ± 11.1

*BMI* Body mass index, *KOA* Knee osteoarthritis, *TUG* Timed up and go test, *CES-D* The Center for Epidemiologic Studies Depression Scale, *PSE* Pain-related self-efficacy, *FSE* Functional self-efficacy, *QOL* Quality of life, *PCS* Physical component summary, *MCS* Mental component summary, *RCS* Role component summary

The mean CES-D-10 score was 5.6 (SD,4.4). According to the criteria, the prevalence of patients with depression was 16.3% (106 patients).

The mean PSE and FSE score were 2.8 (SD,0.8) and 3.4 (SD,0.9), respectively. According to the criteria, the prevalence of participants with high PSE was 59.0% (385) and high FSE was 84.6% (551).

The mean value of three summary scores of SF12 were 41.6 (SD,14.0) in PCS, 50.6 (SD, 10.2) in MCS, and 48.0 (SD, 11.1) in RCS, respectively (Table 1).

### Univariate analysis investigating association between each QOL scores and confounding factors

The influence of each confounding factor to HRQOL was shown in Table 2.

**Table 2:** Univariate analysis investigating association between each QOL scores and confounding factors

	PCS score	*P*-value	MCS score	*P*-value	RCS score	*P*-value
	[mean(SD)]		[mean(SD)]		[mean(SD)]	
age						
< 50	51.2(8.7)	< 0.0001	50.7(7.0)	0.0286	51.1(9.5)	0.0032
50–59	46.1(13.3)		49.3(8.3)		50.9(8.6)	
60–69	44.6(12.1)		49.3(10.0)		49.4(10.6)	
70≦	38.7(14.8)		51.7(10.4)		46.6(11.6)	
gender						
male	44.7(13.0)	< 0.0001	50.7(10.7)	0.8516	49(11.5)	0.074
female	39.9(14.3)		50.6(9.9)		47.4(10.8)	
BMI						
BMI < 25	42.6(13.5)	0.0309	50.5(10.1)	0.6309	47.7(11.1)	0.4987
BMI≧25	40.2(14.8)		50.9(10.4)		48.3(11.1)	
radiographic KOA						
none	46.7(12.0)	< 0.0001	46.0(9.7)	0.0034	49.7(10.4)	0.3708
mild-moderate	44.5(12.9)		50.2(9.8)		48.3(11.4)	
severe	38.0(14.6)		51.6(10.4)		47.4(10.8)	
knee pain						
none	44.7(10.3)	< 0.0001	50.3(11.5)	0.6729	46.1(11.0)	0.1383
mild-moderate	43.6(12.5)		50.5(9.7)		48.4(10.8)	
severe	28.6(16.3)		51.5(12.7)		46.1(12.4)	
TUG(sec)						
TUG < 10	43.4(12.9)	< 0.0001	50.4(9.9)	0.2499	48.9(10.5)	0.0001
TUG≧10	31.8(15.9)		51.7(11.6)		42.9(13.0)	
CES-D-10						
CES-D-10 < 9	42.5(13.4	0.0004	51.7(10.0)	< 0.0001	49.1(10.4)	< 0.0001
CES-D-10≧10	37.2(16.5)		45.2(9.7)		42.3(12.6)	
PSE						
low PSE	35.5(15.2)	< 0.0001	50.8(11.2)	0.7743	44.6(12.0)	< 0.0001
high PSE	45.8(11.4)		50.5(9.4)		50.3(9.8)	
FSE						
low PSE	31.6(15.2)	< 0.0001	51.8(10.8)	0.2194	44(11.5)	0.0001
high PSE	43.4(13.0)		50.4(10.1)		48.7(10.9)	

*SD* Standard deviation, *BMI* Body mass index, *KOA* Knee osteoarthritis, *TUG* Timed up and go test, *CES-D* The Center for Epidemiologic Studies Depression Scale, *PSE* Pain-related self-efficacy, *FSE* Functional self-efficacy

As for the PCS, participants with older age, female gender, higher BMI and grade of radiographic KOA, higher grade of knee pain, decreasing mobility (TUG ≧10 s) were significantly low PCS score compared to those without. In addition, psychological factors also affect PCS. The participants with depression (CES-D-10 ≧10), low PSE, and low FSE had significantly lower score compared to those without.

Participants with depression had lower MCS scores than those without depression. Furthermore, younger participants and those with no or lower-grade radiographic KOA had significantly lower MCS scores than those with older age and high-grade radiographic KOA.

Regarding the RCS score, older age, decreased mobility, depression, and low PSE and FSE had a negative effect on RCS, whereas the severity of radiographic KOA and knee pain did not influence RCS.

### Influencing factors of HRQOL in KOA in multivariable analysis

**Fig. 2:**
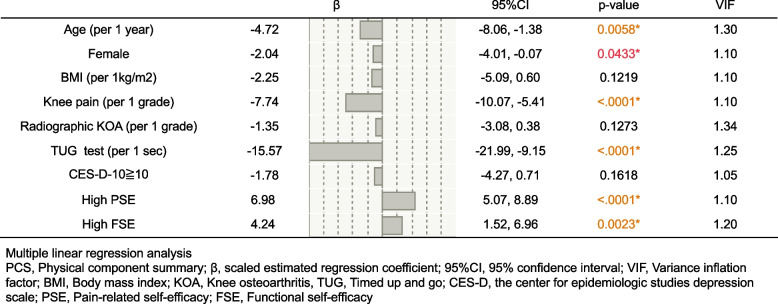
Multiple linear regression analysis investigating the influencing factors of the PCS. Decreasing mobility capability (TUG test) had highest negative effect (β: -15.47). The second was severity of knee pain (β: -7.74), and older age and female gender also had a negative effect to the PCS (β: -4.72 and -2.04, respectively), however, radiographic KOA had no influence on the PCS. Psychological factors also influence PCS. High PSE and FSE had a positive impact on the PCS (β:6.98 and 4.24, respectively), whereas depression had no influence on PCS

As for the PCS, decreasing mobility had the highest negative effect (β: -15.47) (Fig. 2). The second was severity of knee pain (β: -7.74), and older age and female gender also had a negative effect to the PCS (β: -4.72 and -2.04, respectively); however, radiographic KOA had no influence on the PCS. Psychological factors also influence PCS. High PSE and FSE had a positive impact on the PCS (β:6.98 and 4.24, respectively), whereas depression had no influence on PCS.

**Fig. 3:**
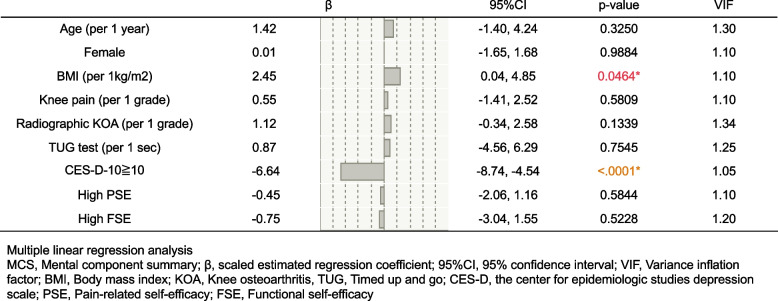
Multiple linear regression analysis investigating the influencing factors of the MCS. The presence of depression had strong negative influence on the MCS (β: -6.64). There were no significant influences in other covariates except for BMI which had a slight positive effect (β:2.45)

Regarding MCS, depression had strong negative influence on the MCS (β: -6.64) (Fig. 3). There were no significant influences in other covariates except for BMI, which had a slight positive effect (β:2.45).

**Fig. 4:**
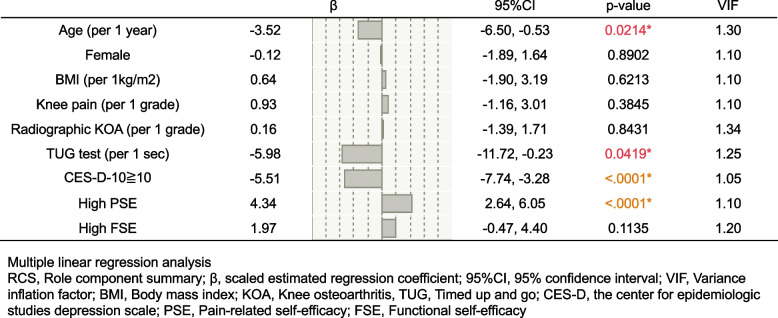
Multiple linear regression analysis investigating the influencing factors of the RCS. Decreasing mobility capacity had highest negative impact to the RCS (β: -5.98). The presence of depression and older age had also negative influence on the RCS (β: -5.51 and -3.52, respectively). As to the self-efficacy, high PSE had significantly positive effect to the RCS (β:4.34). Sex, BMI, knee pain, radiographic KOA, and FSE had no influence on RCS

Decreasing mobility had also the highest negative impact on the RCS (β: -5.98), same as PCS (Fig. 4). The presence of depression and older age had also negative influence on the RCS (β: -5.51 and -3.52, respectively). As to the self-efficacy, high PSE had significantly positive effect to the RCS (β:4.34). Sex, BMI, knee pain, radiographic KOA, and FSE had no influence on RCS. As the VIF of each covariate was quite low in this analysis, there was no multicollinearity between the covariates.

## Discussion

Our study results showed that each component summary score of SF-12 (PCS, MCS, and RCS) was influenced by different confounding factors. Physical QOL was strongly associated with decreasing mobility, severity of knee pain, and pain-related and functional self-efficacy, and mental QOL was mostly influenced by depression. Role/social QOL was predominately affected by age, decreasing mobility, depression, and pain-related self-efficacy. This was the first study referring to the role/social QOL in patients with knee osteoarthritis, and one novelty of this study was that role/social QOL was affected by both physical and psychological factor.

In addition, it was notable that the severity of radiographic KOA had no influence on any of the HRQOL components. It has been widely recognized that the prevalence of knee pain increases with the severity of radiographic KOA; however, several large epidemiological studies have revealed a gap between self-reported knee pain and radiographic severity of KOA [16, 27]. Hannan MT reported that only 47% of moderate to severe knee OA individuals reported knee pain, and only 15% of individuals who reported knee pain had radiographic moderate to severe KOA [16]. Muraki also demonstrated that 57.9% of radiographic grade 2 KOA and 36% of radiographic stage 3 KOA did not experience knee pain [27]. Our study revealed that the presence of radiographic KOA does not necessarily influence pain or HRQOL.

The most important findings of the present study were that high self-efficacy has a positive impact on the HRQOL. Self-efficacy can be defined as a set of beliefs about oneself, specifically about one’s ability to perform certain behaviors in a particular environment [6]. It is considered to be correlated with pain intensity, disability, and daily activity in patients with arthritis, and several reports have described it as a predictor of pain levels and physical functioning in people with chronic pain [3, 4, 9, 25, 34, 37, 38, 40]. Tanaka reported that cognitive characteristics, including pain-related self-efficacy, may predict pain relief in patients with knee osteoarthritis receiving conservative treatment [40]. Large observational register-based study also demonstrated that high self-efficacy had a positive effect on pain and physical activity [9]. In addition, high functional self-efficacy significantly decreased the odds of poor perception of physical functioning and performing poor sit-to-stand activities in people with knee OA [38]. Furthermore, self-efficacy in stair climbing had a moderate relationship with actual stair climbing performance in 480 older adults with knee pain [34]. According to our study, both pain-related and functional self-efficacy were significantly associated with physical QOL as well as the severity of knee pain and the degree of mobility capability. These results suggest that pain control and management of physical functioning, including exploring and strengthening patients’ self-efficacy, are key factors in improving physical QOL.

Our study also showed that high pain-related self-efficacy positively impacted on role/social QOL, as with decreasing mobility, depression and age. The Role/Social Component Summary consists of three of the eight subscales of the SF-12: role physical, social functioning, and role emotional, and reflect the influence of social participation and social engagement that provides individuals with a coherent and consistent sense of role identity, companionship, and sociability. Miles CL described that pain-related self-efficacy in people with pain may include beliefs about one’s ability to control pain and the negative emotions associated with it or to maintain everyday life activities, including work [26]. According to our results, pain-related self-efficacy might play an important role not only in improving personal physical QOL but also in building a smooth relationship with others and society.

It has been reported that depression and anxiety associated with knee pain and activity limitations in the subjects with KOA [8, 17, 18, 20, 25, 35]. Kim et al. described that the presence of a depressive disorder was associated with an increased risk of symptomatic knee osteoarthritis [20], and Hoffa et al. showed that depressed mood was independently associated with knee pain and activity limitations in patients with knee OA [18]. On the other hand, several reports have described that actual physical performance is not affected by depression or other psychological distress [17, 25, 35]. Similar to the previous studies, our study demonstrated that the presence of depression had a negative impact on both mental and role/social QOL, whereas it had no influence on physical QOL. According to these findings, controlling the psychological condition might be important to organize patients’ healthy social lives as well as to maintain their mental well-being.

This study had some limitations. First, there may have been a selection bias because the participants voluntarily attended the health check-ups as well as X-ray examination. Relatively healthy and health-conscious individuals may have participated in this study.

Second, the area surveyed in this study is a rural area in Japan with a particularly aged population. Similar epidemiological studies should be conducted in urban areas to eliminate regional differences and enhance the reliability of statistical analyses. Third, there was a possibility that the scale was insufficient to determine the level of self-efficacy because we selected only two questions from the original version of the ASES. In addition, a validation study was not done for these modified questions. Therefore, it is necessary to use a full scale to evaluate the precise level of self-efficacy. Finally, because our study was cross-sectional, it was not possible to assess the causal relationship between HRQOL and confounding factors. A prospective cohort study should be conducted to clarify the causal relationship between HRQOL and confounding factors.

## Conclusions

Our results showed that physical, mental, and role/social QOL were affected by different confounding factors. Physical QOL was strongly influenced by subjective pain, physical performance, and self-efficacy, whereas radiographic KOA score had no such effect. Depressive mood is associated with both mental and role/social QOL. The role/social QOL was predominantly affected by physical function and pain-related self-efficacy. Taking measure to improving functional ability and mental status might be the key factor to improve HRQOL in patient with knee osteoarthritis.
